# Three-Dimensional Mapping of Shear Wave Velocity in Human Tendon: A Proof of Concept Study

**DOI:** 10.3390/s21051655

**Published:** 2021-02-27

**Authors:** Tobias Götschi, Nicole Schulz, Jess G. Snedeker, Jonas Hanimann, Martino V. Franchi, Jörg Spörri

**Affiliations:** 1 Department of Orthopaedics, Balgrist University Hospital, University of Zurich, 8008 Zurich, Switzerland; jess.snedeker@hest.ethz.ch; 2 Institute for Biomechanics, ETH Zurich, 8093 Zurich, Switzerland; schulzn@student.ethz.ch; 3 Sports Medical Research Group, Department of Orthopaedics, Balgrist University Hospital, University of Zurich, 8008 Zurich, Switzerland; jonashan@student.ethz.ch (J.H.); martino.franchi@unipd.it (M.V.F.); Joerg.Spoerri@balgrist.ch (J.S.); 4 Institute of Physiology, Department of Biomedical Sciences, University of Padova, 35121 Padova, Italy; 5 University Centre for Prevention and Sports Medicine, Department of Orthopaedics, Balgrist University Hospital, University of Zurich, 8008 Zurich, Switzerland

**Keywords:** imaging, biomechanics, ultrasound, shear wave elastography, stereophotogrammetry, tendon, tendinopathy, validation study, reproducibility of results

## Abstract

Ultrasound-based shear wave elastography (SWE) provides the means to quantify tissue mechanical properties in vivo and has proven valuable in detecting degenerative processes in tendons. Its current mode of use is for two-dimensional rendering measurements, which are highly position-dependent. We therefore propose an approach to create a volumetric reconstruction of the mechano-acoustic properties of a structure of interest based on optically tracking the ultrasound probe during free-hand measurement sweeps. In the current work, we aimed (1) to assess the technical feasibility of the three-dimensional mapping of unidirectional shear wave velocity (SWV), (2) to evaluate the possible artefacts associated with hand-held image acquisition, (3) to investigate the reproducibility of the proposed technique, and (4) to study the potential of this method in detecting local adaptations in a longitudinal study setting. Operative and technical feasibility as well as potential artefacts associated with hand-held image acquisition were studied on a synthetic phantom containing discrete targets of known mechanical properties. Measurement reproducibility was assessed based on inter-day and inter-reader scans of the patellar, Achilles, and supraspinatus tendon of ten healthy volunteers and was compared to traditional two-dimensional image acquisition. The potential of this method in detecting local adaptations was studied by testing the effect of short-term voluntary isometric loading history on SWV along the tendon long axis. The suggested approach was technically feasible and reproducible, with a moderate to very good reliability and a standard error of measurement in the range of 0.300–0.591 m/s for the three assessed tendons at the two test-retest modalities. We found a consistent variation in SWV along the longitudinal axis of each tendon, and isometric loading resulted in regional increases in SWV in the patellar and Achilles tendons. The proposed method outperforms traditional two-dimensional measurement with regards to reproducibility and may prove valuable in the objective assessment of pathological tendon changes.

## 1. Introduction

Tendon-related complaints, such as tendinopathy, are common in athletes, workers, and the general population [1], with the patellar, Achilles, and supraspinatus tendons being among the most frequently affected [2]. The typical diagnostic procedure of tendinopathies includes the reporting of symptoms during daily activities, the manual palpation of the tendon and its insertions (pressure-induced pain), as well as radiological signs through imaging. Additionally, quantitative assessments of the elastic properties of tendon structures have proven valuable in identifying pathologic and traumatic conditions [3]. Degenerative processes of such conditions include an increase in collagen type III fibers, fibrocartilaginous changes caused by an upregulated production of glycosaminoglycans (GAGs), tenocyte rounding and proliferation, and neovascularization [4,5,6,7]. Collagen type III fibers exhibit a reduced ability to form cross-links compared to collagen type I fibers, and their accumulation results in reduced fiber orientation [5]. Moreover, GAGs contain highly hydrophilic side chains and increase the water content in the tendon [8,9]. As a consequence of these adaptations, the stiffness of pathological tendons is reduced [10,11,12], favoring traumatic tendon injuries such as ruptures [13,14].

Ultrasound (US) shear wave elastography (SWE) allows a quantitative assessment of local tissue elasticity. Briefly, a focused acoustic radiation force impulse displacing the tissue is produced. This tissue displacement propagates perpendicularly to the direction of the impulse as a shear wave, which can be observed with a high frame rate (3–18 kHz) brightness-mode (B-mode) US and appropriate tracking algorithms [15]. The instantaneous group velocity of such shear waves is related to the tissues’ elastic properties and can be mapped on a regular grid superimposed onto the B-mode US image [16]. Quantitative measurements of the elastic properties of structures of the musculoskeletal system have proven valuable in identifying various pathologic and traumatic conditions [3]. In particular, SWE has the potential to depict tendon damage and degeneration and predict impending structural failure [17].

Commonly, when investigating specific tissue properties using US SWE, the scans are performed in a two-dimensional (2D) fashion with a limited field of view (FOV), severely complicating the assessment of larger structures and rendering the retrieved results highly position-dependent. Specifically in the case of tendon imaging, pathological alterations in structural composition and architecture are oftentimes spatially confined and their assessment over time consequently requires accurate spatial referencing.

One potential solution to overcome these limitations might be found in the following approach: when the US transducer’s position and orientation (i.e., its pose) over a series of measurement frames is known, 2D measurements can be projected into three-dimensional (3D) space, allowing the volumetric reconstruction of shear wave velocity (SWV) and the underlying B-mode images. To this end, we propose a free-hand 3D ultrasound approach based on optical probe tracking on a SWE-capable US device in order to obtain a 3D mapping of unidirectional shear wave velocity of human tendons in vivo, hereafter called 3D SWVM. Such an approach enables the investigation of larger structures, only limited by the maximum measurement depth of the device (approximately 5 cm [18]), and analyses of substructures can be performed offline. As for any new measurement technique, criteria of sufficient validity and reproducibility must be met in order to provide a clinically viable assessment tool. Moreover, particularly in the context of intra-subject effects in a longitudinal setting, the ideal technique should enable the investigation of tendon adaptation with sufficient spatial resolution.

Therefore, the aims of the study were: (1) to assess the technical feasibility and validity of 3D SWVM by scanning an elastography tissue phantom and validating the acquired data with the reference values of the substructures in the phantom provided by the manufacturer; (2) to evaluate potential artefacts caused by out-of-plane transducer motion during image acquisition at varying transducer speeds; (3) to investigate the inter-operator- and inter-day reproducibility of 3D SWVM in patellar-, supraspinatus-, and Achilles tendons compared to traditional 2D SWVM; and (4) to assess the effect of isometric loading on local tendon SWV in healthy adult subjects.

## 2. Materials and Methods

### 2.1. Free-Hand 3D Shear Wave Velocity Mapping

Measurements of shear wave group velocity were acquired as provided by the ultrasound device (Aixplorer Ultimate, SuperSonic Imagine, Aix-en-Provence, France) using a linear 5 cm transducer (SuperLinear SL18-5, SuperSonic Imagine, Aix-en-Provence, France). In order to project 2D US frames into 3D space, the transducer was equipped with a custom-built marker set and image positions and orientations were recorded synchronous to image acquisition with an optical tracking system (FusionTrack 500, Atracsys LLC, 7 Hz sampling frequency, tracking accuracy 0.09 mm (RMS)). The volumetric sampling of the volume of interest was achieved by manually moving the US transducer orthogonal to the imaging plane in a continuous motion (<5 mm/s). Where the volume of interest was larger than the lateral FOV, the transducer was repositioned during the scanning procedure with the image acquisition halted. In vivo measurements consisted of 3 of these measurement sweeps, whereas phantom measurements were acquired in one continuous motion. The volumetric reconstruction of both B-mode and SWV data was performed using a voxel-based approach averaging pixel intensities, where multiple pixels were sampled into the same voxel at an isometric resolution of 0.5 mm [19,20]. For the reconstruction of SWV volumes, pixel values of zero were discarded before reconstruction because these do not constitute valid SWV estimates [3]. Depending on the transducer path during scanning, multiple frames map into the same voxel. The overall SWV ($\bar{v}$) of a structure was therefore estimated by using a weighted mean of all voxels contained within the segmentation, weighting ($\omega_{i}$) each voxel’s ($V_{i}$) average SWV ($v\left(V_{i}\right)$) with the inverse of its respective standard error:

(1) $$\bar{v}=\frac{\sum_{i=1}^{n}\omega_{i}\times v\left(V_{i}\right)}{\sum_{i=1}^{n}\omega_{i}},\;with\;\omega_{i}=\;\frac{\sqrt{n\left(V_{i}\right)}}{\sigma \left(V_{i}\right)}$$

To prevent the occurrence of singularities, the minimum of $\sigma \left(V_{i}\right)$ was set to be 0.5 m/s. B-mode images were acquired with a sampling frequency of 7 Hz. SWE images can be acquired at a maximum frequency of 2 Hz. One dataset typically consisted of a total of 1000 B-mode and 200 SWE US images. Images were recorded and processed using MATLAB (2019b, The MathWorks, Inc., Natick, MA, USA). Volumetric segmentation was performed manually by using the underlying 3D B-mode reconstruction [21].

### 2.2. Phantom Experiments

Technical feasibility and validity of free-hand 3D SWVM was assessed using an elastography tissue phantom (Elasticity QA Phantom, model 049, CIRS Inc., Virginia, WV, USA) containing four different types of acoustic inclusions of uniform known stiffness. 3D SWVM was performed by scanning each inclusion separately with the transducer oriented perpendicular to the long axis of the inclusion. A total of 200 SWS frames were obtained per measurement, and each measurement was repeated three times. 2D SWVM was performed by positioning the transducer statically over the largest cylinder of the inclusion with analogous transducer orientation. The 2D measurement was repeated five times and each measurement was segmented manually.

To determine the effect of out-of-plane transducer motion on shear wave velocity estimates, the US transducer was connected to the crosshead of a materials testing machine (ZWICK Roell Z010, Ulm, Germany) in order to be able to accurately manipulate the speed at which the phantom was scanned. 3D SWVM was performed of the largest cylinder of each inclusion applying a constant transducer speed of motion and repeating the measurement at varying transducer speeds ranging from 0 to 33 mm/s, with the latter comprising the maximum applicable crosshead speed of the machine. Each measurement consisted of 125 SWV frames. The mean SWV of the inclusion was calculated as described in Equation (1). Young’s modulus (E) values of the inclusions provided by the manufacturer were converted into shear wave velocity (v, ms^−1^), with an assumed density (ρ) of 1040, 1040, 1050, and 1060 kgm^−3^ for the inclusion of type I to IV [22], respectively, based on the following equation [23]:

(2) $$v=\;\sqrt{\frac{E}{3\rho }}$$

### 2.3. In Vivo Reproducibility Experiments

Ten healthy subjects (mean (SD) age: 28.1 (3.0), female: 4) without history of upper or lower limb musculoskeletal pathology were included and the right patellar, Achilles, and supraspinatus tendon were imaged. All the subjects reported to be physically active (at least one intense or more than 30 min of moderate physical activity per week). The in vivo study was approved by the local ethics committee (KEK-ZH-NR: 2017-01395). All the participants were informed in writing about the measurement procedures and provided written consent.

All the tendon measurements were conducted with the US transducer oriented parallel to the fiber axis of the tendon, which can be accomplished by observing and maximizing the fibrillar appearance of the intratendinous structure in relation to transducer rotation (about its long axis). Minimal transducer pressure was applied, and proper acoustic transmission was ensured with the application of a generous amount of US gel. The subjects were positioned at least 5 min prior to the assessment to prevent any immediate loading history from affecting the measurements and were instructed to remain as relaxed as possible, during this preconditioning phase as well as during the scanning procedure. Each structure was imaged by both operators on day one in a randomized order. Approximately 24 h later, each structure was imaged twice by operator one, once to assess inter-day reproducibility and a second time immediately following a 15 s maximum voluntary isometric tendon loading exposure. Post-loading measurements were conducted with the subjects in the same position as during pre-loading measurements. The experiment was powered to yield an intra-class correlation (ICC) precision of ±0.15 to detect a pre-post loading effect of Cohen’s d = 1 [24].

#### 2.3.1. In Vivo 3D SWVM

**Figure 1:**
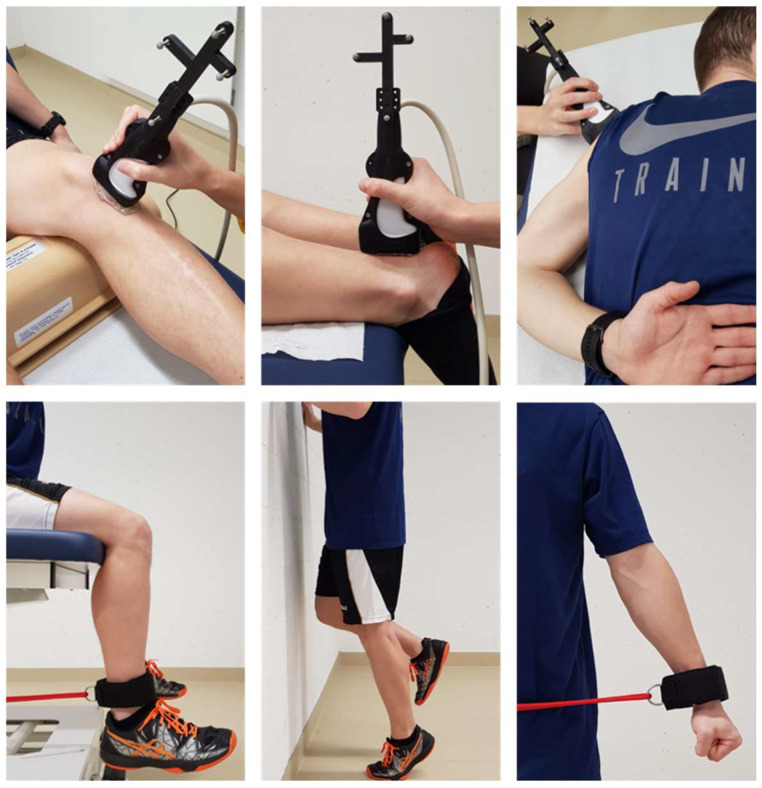
**Top** row: Subject position during ultrasound examination of the patellar, Achilles, and supraspinatus tendon, respectively. **Bottom** row: experimental setup used to induce tendon loading stimulus for either of the three tendons assessed.

Figure 1 provides an illustration of the subject positioning during US examination and isometric tendon loading.

The patellar tendon was imaged with the subject in supine position and an adjustable support underneath the knee with a knee flexion angle of approximately 20 degrees. 3D SWVM was performed on the entire tendon length. For 2D SWVM, the transducer was positioned over the thickest part of the tendon parallel to its long axis, ensuring the region of interest (ROI) as described below is fully included. The tendon loading exposure consisted of having the subject in a sitting position with the knees flexed 90 degrees, the right ankle fixated by a brace, and instructing the subject to perform a 15 s maximum voluntary isometric knee extension.

For the imaging of the Achilles tendon, the subject was lying prone on an examination table with their foot hanging just over the edge, ensuring a relaxed ankle position. 3D SWVM was performed on the distal portion of the tendon for a total length of approximately 10 cm. For the tendon loading exposure, the subject was instructed to perform a one-legged complete foot plantarflexion in a standing position, touching the wall for balance.

The imaging of the supraspinatus tendon was performed with the subject in supine position with the right arm positioned under the lumbar back with the palm facing down. Tendon loading consisted of brace-resisted arm abduction with the elbow joint in extension.

#### 2.3.2. In Vivo 2D SWVM

**Figure 2:**
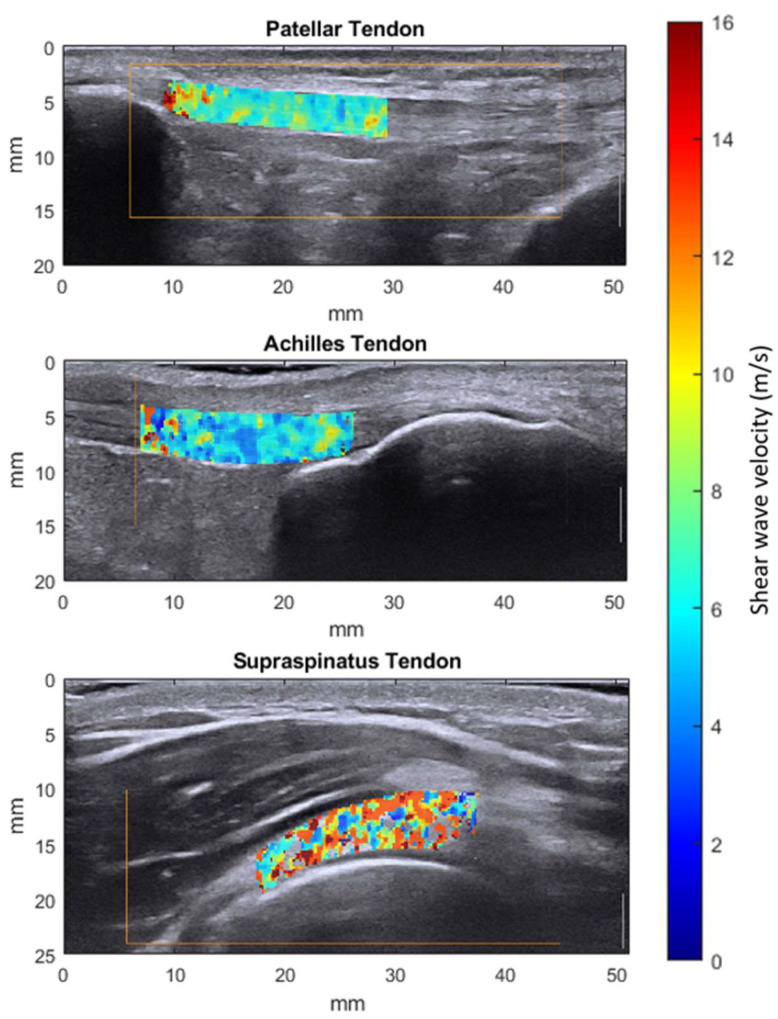
Exemplary 2D shear wave elastography measurements of the 3 types of tendons studied after the manual segmentation of the region of interest.

During the in vivo assessments, six 2D SWVM measurements were acquired by positioning the transducer statically over the central portion of the tendon, oriented parallel to its long axis. The region of interest (ROI) was defined as a full-thickness portion of the tendon with a length of 2 cm and was segmented manually post-measurement. In the patellar tendon, the proximal border of the ROI was defined as the patellar tendon insertion. The ROI for the Achilles and the supraspinatus tendon was placed just proximal to the tendon insertion (Figure 2). The mean shear wave velocity for one measurement sequence was then calculated by averaging all the pixel values of the six frames contained in the segmentation mask.

#### 2.3.3. Tendon Response to Loading

SWV as a function of relative longitudinal position along the axis of the tendon before and after the loading exposure was computed. In the three tendon types, the following registration landmarks were defined: patellar tendon: the most proximal point of tibial insertion and the most distal point of patellar insertion; Achilles tendon: the most proximal point of calcaneal insertion and proximal location with a distance of 10 cm from the distal tendon-calcaneus insertion; supraspinatus tendon: the most distal and most proximal point of humerus insertion (see the illustrations in the top row of Figure 6). These landmarks were manually annotated and used to orient the volumes and were kept at fixed longitudinal positions in order to obtain a positional frame of reference. Averaging along the longitudinal axis was performed using a Gaussian convolution kernel (σ = 5 mm) and the voxel weighting scheme described in Equation (1).

### 2.4. Statistical Analysis

The validity of both measurement modalities was assessed by comparing the mean SWV of each phantom inclusion, averaged over the three test repetitions with the manufacturer-provided reference values. Measurement reproducibility was analyzed in the two domains of measurement agreement and measurement reliability. The prior was described in terms of standard error of measurement (SEm), and the latter was described using intra-class correlation coefficients (ICC(2, 1)) [25] and associated 95% confidence intervals based on a two-way random effects model assessing the absolute agreement of a single-measure approach [26]. ICC values were classified as poor (≤0.2), fair (0.21–0.4), moderate (0.41–0.6), good (0.61–0.8), and very good (>0.8) [27]. The effect of tendon loading on the overall SWV was assessed with a paired-samples t-test. Analogously, paired-samples t-tests were performed on each computed longitudinal position to test for regional loading effects. Statistical analysis was conducted with MATLAB (2019, The MathWorks, Inc., Natick, MA, USA) and SPSS (Version 26, IBM Corp., Armonk, NY, USA). Statistical significance was set at α = 0.05.

## 3. Results

### 3.1. Elastography Phantom

**Figure 3:**
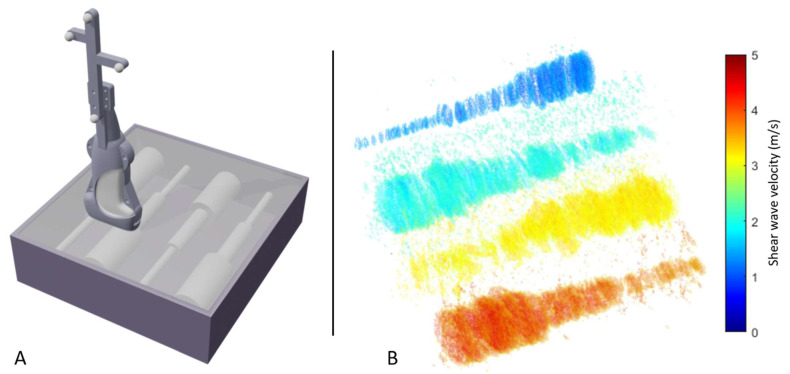
Elastography phantom scan. (**A**) Schematic depiction of the procedure used to validate 3D shear wave velocity on an elastography phantom including discrete targets of known stiffness. The phantom was scanned with the US transducer oriented orthogonal to the contained cylinders and volumetric mapping was implemented using a motion capture system and a reflective marker set attached to the transducer. (**B**) Heatmap visualization of the volumetric shear wave velocity reconstruction of the four inclusions.

Scanning the elastography phantom revealed 3D SWVM to be technically feasible. Figure 3 presents a visualization of the reconstruction. Each voxel’s opacity was determined based on its assigned shear wave velocity with varying thresholds for the different phantom inclusion types. The shape of the phantom inclusions can readily be distinguished solely based on the stiffness information available.

The calculated mean shear wave velocities of the four phantom inclusions using 3D SWVM and 2D SWVM were subsequently compared with the reference values provided by the manufacturer. 3D SWVM underestimated the reference values by −13.4%, −18.4%, −23.5%, and −24.0%, whereas 2D SWVM showed accuracies of −10.1%, −20.2%, −22.6%, and −23.7% related to the four types of inclusions, respectively (Table 1).

**Figure 4:**
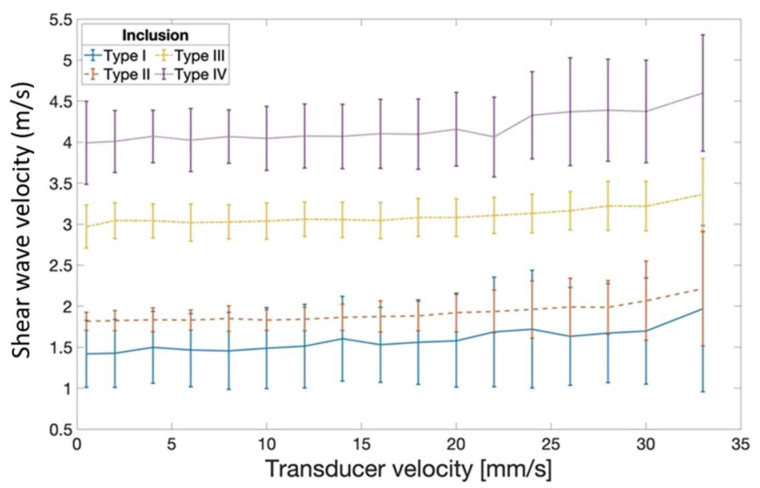
Mean shear wave velocity estimates based on 3D SWVM as a function of the speed of out-of-plane transducer motion during measurement. Estimates remain relatively robust at transducer motion below 20 mm/s. Error bar: ±SD.

In a separate experiment, the influence of transducer motion during measurement was quantified. When compared to transducer-static recordings, transducer motion below 20 mm/s introduced a maximum measurement error of 0.15 m/s during the scanning of inclusion type IV (Figure 4). Varying transducer motion up to 33 mm/s yielded a persistent SWV overestimation compared to the velocity values acquired during static recording.

3D SWVM yielded a high reproducibility for elastography phantom measurements with an ICC = 0.999 (0.987; 1) and SEm = 0.043 m/s. Similarly, 2D SWVM yielded an estimated ICC of 0.993 (0.970; 0.999) and a SEm of 0.10 m/s.

### 3.2. In Vivo Tendon Imaging

**Figure 5:**
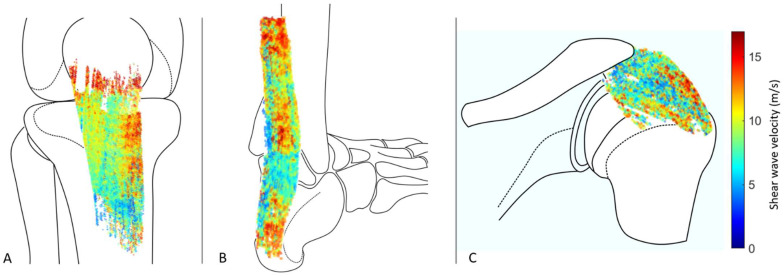
Heatmap visualization of 3D SWVM of the patellar (**A**), Achilles (**B**), and supraspinatus (**C**) tendon after manual segmentation. Artistic representations of the bony structures are added for more clarity.

3D B-mode reconstruction allowed the manual segmentation and subsequent masking of the 3D SWVMs of the acquired in vivo measurements (Figure 5).

The reliability of the in vivo 3D SWVM of tendons ranged between moderate to very good, whereas the measurement uncertainty was between 0.303 and 0.591 m/s (Table 2). 2D SWVM, on the other hand, displayed a poor to moderate reliability, with an SEm between 0.516 and 1.068 m/s (Table 3).

### 3.3. Tendon Response to Loading

**Figure 6:**
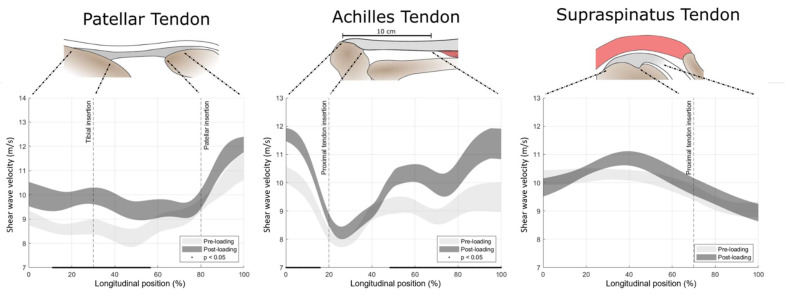
**Top** row: schematic representation of the assessed structures with the anatomical landmarks used for spatial referencing. **Bottom** row: areas of uncertainty around the estimate of the mean (±SE) shear wave velocity before and after 15 s maximum voluntary isometric tendon loading over the longitudinal axis of the tendon. Longitudinal positions with a statistically significant load response are underlined with a black line.

Maximum voluntary isometric tendon loading resulted in an overall mean (±SD) increase in the SWV of 0.70 ± 1.16 (*p* = 0.090), 0.75 ± 0.81 (*p* = 0.017), and 0.04 ± 0.92 m/s (*p* = 0.888) in the patellar, Achilles, and supraspinatus tendon, respectively. The analysis of SWV along the longitudinal axis of the tendon revealed considerable regional variation. Likewise, the response to loading was highly position-dependent, with a significant increase in the SWV for specific regions in the patellar and Achilles tendons (Figure 6).

## 4. Discussion

In the current study, we found (1) 3D SWVM to be technically feasible with a high reproducibility in elastography phantom measurements, and with similar bias as the traditional 2D SWVM approach when compared to the manufacturer-provided reference values. (2) Out-of-plane transducer motion had a small nonlinear effect on the SWV estimates, which may be considered negligible in most potential applications. (3) In vivo 3D SWVM showed moderate to good reliability for the Achilles and supraspinatus tendons, whereas for the patellar tendon it was shown to have a good to very good reliability. The reliability of 2D assessments on the other hand ranged from poor to moderate. (4) There was considerable variation in SWV along the longitudinal axis of all tendons, with significant increases in SWV after tendon loading in specific regions of the patellar and the Achilles tendons.

### 4.1. Three-Dimensional Mapping of Shear Wave Velocity in Human Tendon Is a Technically Feasible and Valid Approach

Within the elastography tissue phantom experiment, the technical feasibility of generating a 3D map of SWV was clearly demonstrated. We further note that the assessment time and operator proficiency required to robustly acquire images with sufficient technical quality were compatible with the eventual clinical translation. The random error was revealed to be sufficiently small. However, the validation of the absolute values against the manufacturer-provided reference values yielded only limited agreement. The elastic response of most materials is dependent on the strain as well as the rate at which it is applied. Moreover, the depth of measurement [28] as well as the size of the assessed inclusion [29] has been reported to influence results. Mulabecirovic et al. have reported better agreement with manufacturer-provided reference values when using traditional 2D SWVM [22]. A possible explanation for this discrepancy is that this study limited analysis to the central region of the phantom inclusions, whereas in the current study the inclusion was analyzed in its entire diameter.

### 4.2. Potential Artefacts Caused by Out-of-Plane Transducer Motion during Image Acquisition at Varying Transducer Speeds

When using conventional SWE imaging, the transducer is usually held stationary during the measurement process, which is not possible when scanning an entire tissue volume. Out-of-plane transducer motion introduces various potential sources of additional measurement error, such as biased tissue strain estimation due to changes in the imaged scatterer configuration during SW tracking. Current SWE devices rely on plane-wave B-mode imaging at frame rates exceeding 5000 Hz, and tissue deformation is usually estimated merely from one frame to the next [30]. Consequently, at a transducer speed of motion of 33 mm/s the out-of-plane displacement of the FOV between two consecutive measurements will lie below 0.01 mm. This miniscule displacement evidently does not prohibit measurement but may conceivably reduce the signal-to-noise ratio in the strain field maps. The current results indicate a nonlinear influence of out-of-plane transducer motion on SWV estimates. When the transducer motion is kept below 20 mm/s however, the maximum measurement error of 0.15 m/s may be rated acceptable for most musculoskeletal applications. The in-plane transducer motion had no effect on SWV measurements in a previous study [31].

### 4.3. Inter-Operator and Inter-Day Reproducibility of 3D SWVM in Human Tendon

The reproducibility of a measurement can be described in two distinct domains often denoted as agreement and reliability. Whereas the prior aims to determine the measurement error inherent to the technique under investigation, the latter relates test–retest discrepancies (intra-subject variability) to the overall spread of the acquired data and is consequently a direct function of the heterogeneity of the studied subject cohort [26]. In view of the fact that the current cohort was comprised of healthy, physically active subjects with a narrow age range, the presented ICC values are a conservative estimate of the reliability to be expected in a cohort more representative of the general population [32,33,34], and in particular when including cases with symptomatic tendons [35].

The technique under study yielded highly reproducible measurements in the controlled laboratory setting, with a marked decrease in reproducibility for in vivo measurements, which can be attributed to multiple potential factors. The tendon is composed of primarily aligned collagen, and therefore SWV is orientation-dependent [34,36]. Indeed, Peltz et al. [37] reported a lower reliability of ICC = 0.85 in an in vitro tendon SWE study. Tendon SWV is position-dependent [33,35,38], which may explain the lower reproducibility in the case of 2D SWE. Furthermore, tendon elasticity undergoes natural fluctuations in relation to loading history [39] and hydration state [40].

The measurement reproducibility of 2D SWVM for the assessment of either tendon studied herein has been described previously with varying results. A number of those investigations studied a cohort with a high inherent heterogeneity either by reporting pooled reliability for multiple age groups [34] or by combining healthy subjects and subjects with a relevant pathology [41], rendering direct comparison difficult. Others based their analysis on estimates of Young’s modulus rather than analyzing the underlying SWV [33,42,43,44,45,46]. We chose to analyze the latter, since the simple conversion formula shown in Equation (2) relies on material isotropy and linear elasticity, assumptions clearly violated in the case of tendons. Other conversion approaches are not straightforward and material model-dependent [47]. A further factor limiting comparability with some studies [36,48,49] is patient positioning and the imposed joint flexion angle during measurement. Among other factors, tendon SWV is directly dependent on the tensile force during measurement [46,50]. For instance, Baumer et al. [48] reported an increase in the inter-day reliability of supraspinatus tendon measurements when the subject was actively lifting his arm (ICC = 0.94) compared to the relaxed position (ICC = 0.48). In the context of assessing tendon integrity, SWE aims to describe characteristics inherent to the material, rather than being a proxy for tendon loading; since the weight of the arm will vary (reproducibly) among the subjects, the prior protocol may yield critically biased results.

The available literature indicates the 2D SWVM of the patellar tendon to be region-dependent, with ICC values in the range of 0.71–0.83 [35,51] and 0.40–0.84 [49,51] in an inter-day and inter-operator setting, respectively. Achilles tendon assessments were reported to yield an inter-day reliability of ICC: 0.54–0.71 [35,52], and an inter-operator reliability of ICC = −0.01 with the foot in neutral position [36]. Regarding the reproducibility of supraspinatus tendon measurements, apart from the study performed by Baumer et al. mentioned above, we did not find any additional literature allowing direct comparison to the current study. Few of the mentioned reproducibility studies quantify measurement agreement. Tas et al. [53] estimates the SEm for patellar tendon 2D SWVM to be 0.51–0.56 and 0.7 m/s, in an inter-day and inter-operator setting, respectively. Payne et al. [52] reports a high inter-day agreement with SEm = 0.23 m/s for Achilles tendon assessments with a relaxed foot position.

### 4.4. Local Tendon Shear Wave Velocity

We found consistent variation in SWV along the longitudinal axis of each tendon. Such behavior was not present along the medio-lateral or the superficial-deep axis (data not shown). The observed variation is likely a result of an interplay of various factors. Tendon tissue changes towards both the muscular and the bony transition in structure and composition [54,55,56]. Moreover, guided shear wave propagation (leaky Lamb wave guided mode) may cause the SWV to be dependent on the tendon diameter [47]. Irrespective of the nature of the underlying factors, the observed position-dependency underlines the necessity of accurate spatial referencing of SWE measurements both in intra- as well as in inter-subject study settings.

### 4.5. Effect of Isometric Loading on Tendon Shear Wave Velocity in Healthy Adult Subjects

Tendons’ biochemical, biomechanical, and structural properties adapt in response to loading or the absence thereof in the long term. Towards a deeper understanding of the mechanisms governing chronic tendon adaptation, the study of acute effects of loading may be pivotal [57]. In our experiment, all three tendon structures behaved consistently with an increase in SWV in response to 15 s maximum voluntary isometric contraction, with statistical significance only for the patellar and the Achilles tendon. Data from the literature on the matter are sparse but support our findings in that variable modes of loading resulted in an increase in Achilles tendon SWV [39,44,45]. Contrary to these findings, the global tendon stiffness assessed by measuring force-controlled tendon elongation was reduced following isometric contractions [58] and static stretching [59] in the Achilles tendon, as well as following eccentric knee extension exercises in the patellar tendon [60]. Structurally, tendon loading has been reported to induce the uncrimping and re-alignment of wavy collagen fibers and reduce fluid content [61,62]. These changes conceivably lead to reduced hysteresis as a result of an increase in the capacity for elastic energy storage accompanied by reduced viscosity [63]. According to theoretical modelling, however, longitudinal tendon SWV is not affected by viscous behavior, which may explain this apparent discrepancy of increased SWV at lower global tendon stiffness [47]. Following this line of thought, a tendon’s response to loading in SWV may be indicative of its structural organization and could potentially be used as a marker for the presence of functionally impaired tissue, such as that found in partial tendon rupture and degeneration [4], and warrants further investigation.

### 4.6. Methodological Considerations

There are limitations to this study that need to be considered. The current investigation solely assessed reproducibility related to the imaging procedure. Inter-evaluator differences during the manual segmentation of the acquired data would additionally have to be considered before the large-scale implementation of the technique. As evident from the large variation in SWV across the tendon long axis, depicting the entire measurement consisting of millions of localized SWV estimates in one value may be overly simplistic in clinical applications, but we believe this serves its purpose in establishing measurement reproducibility.

## 5. Conclusions

In the current study, we found the 3D mapping of unidirectional shear wave velocity to be a feasible extension of traditional 2D measurement with improved reproducibility. The volumetric spatial referencing of the measurements in a longitudinal study setting allows the objective characterization of local alterations in mechano-acoustic properties and demonstrates potential towards the functional assessment of both pathological and physiological tendon adaptations.

## Figures and Tables

**Table 1:** Mean (±SD) shear wave velocity of the four inclusion types in the elastography tissue phantom. Calculated mean values in the reconstructed 3D volume (3D SWVM) and 2D images (2D SWVM) with respective standard deviations in comparison to the reference values provided by the manufacturer.

Inclusion	3D SWVM [m/s]		2D SWVM [m/s]		Reference Values by Manufacturer [m/s]
Type I	1.27	(±0.05)	1.32	(±0.22)	1.47
Type II	1.83	(±0.04)	1.79	(±0.02)	2.24
Type III	3.09	(±0.00)	3.13	(±0.03)	4.04
Type IV	4.12	(±0.07)	4.13	(±0.03)	5.42

**Table 2:** Estimates of intra-class correlation coefficients (ICC) and standard error of measurement (SEm) for the assessment of the patellar, Achilles, and supraspinatus tendons using 3D shear wave velocity mapping.

3D SWVM	Inter-Operator		Inter-Day	
	ICC (95% CI)	SEm [m/s]	ICC (95% CI)	SEm [m/s]
Patellar tendon	0.736 (0.270; 0.926)	0.440	0.904 (0.680; 0.975)	0.303
Achilles tendon	0.436 (−0.195; 0.820)	0.553	0.591 (−0.015; 0.878)	0.505
Supraspinatus tendon	0.632 (0.079; 0.892)	0.591	0.556 (−0.037; 0.866)	0.501

**Table 3:** Estimates of intra-class correlation coefficients (ICC) and standard error of measurement (SEm) for the assessment of the patellar, Achilles, and supraspinatus tendons using 2D shear wave velocity mapping.

2D SWVM	Inter-Operator		Inter-Day	
	ICC (95% CI)	SEm [m/s]	ICC (95% CI)	SEm [m/s]
Patellar tendon	0.495 (−0.122; 0.842)	0.892	0.545 (−0.053; 0.862)	0.901
Achilles tendon	0.455 (−0.172; 0.827)	1.043	0.591 (0.014; 0.878)	1.068
Supraspinatus tendon	0.350 (−0.291; 0.783)	0.761	0.323 (−0.318; 0.772)	0.516

## Data Availability

The data supporting the reported results can be found at https://doi.org/10.6084/m9.figshare.14124152.v2.
